# Body Size and Local Density Explain Movement Patterns in Stream Fishes

**DOI:** 10.1002/ece3.72996

**Published:** 2026-02-11

**Authors:** Ashley LaRoque, Seoghyun Kim, Akira Terui

**Affiliations:** ^1^ Depatment of Biology University of North Carolina at Greensboro Greensboro North Carolina USA; ^2^ Department of Biological Sciences Kangwon National University Chuncheon South Korea

**Keywords:** body size, density‐dependent, extrinsic and intrinsic, fish movement, freshwater, movement drivers

## Abstract

Movement is a fundamental process in structuring communities, distributing species, and mediating gene flow. Both extrinsic (e.g., density of species) and intrinsic factors (e.g., body size) influence movement patterns, ultimately driving the spatial organization of ecological communities. However, these extrinsic and intrinsic factors are often assessed in isolation, limiting our ability to understand how multiple factors combine to shape movement patterns in nature. Here, we evaluate whether body size (intrinsic) and intra‐ and interspecific densities (extrinsic) have an impact on the movement rates of four fish species ( *Nocomis leptocephalus* bluehead chub, *Semotilus atromaculatus* creek chub, *Lepomis cyanellus* green sunfish, and *L. auritus* redbreast sunfish) in a small stream. We employed a capture‐mark‐recapture framework to individually track movements, defined as the difference between locations on consecutive (re)captures. We then applied a dispersal‐observation model that accounts for detectability, survival, and emigration when inferring movement processes. We found that larger individuals of creek chub and green sunfish were more likely to move, which may be explained by their greater physical ability to balance the energetic cost of moving in tandem with greater competitive ability during settlement. The effect of density on movement was mixed. Green sunfish moved away from areas with high density of creek chub, but movement declined when bluehead chub density was high. Bluehead chub responded reciprocally to green sunfish, with less movement at high green sunfish density. Movement also declined for creek chubs in the presence of bluehead chub. This may suggest that certain species interact due to predator–prey interactions either directly or indirectly. Collectively, our results suggest that intrinsic (body size) and extrinsic factors (density) influence movement patterns, but their relative importance is species‐specific. Further exploring the mechanistic relationship behind drivers of movement will provide greater insights into spatial community dynamics.

## Graphical abstract

We evaluated whether body size (intrinsic) and intra‐ and interspecific densities (extrinsic) have an impact on the movement rates of four fish species in a small stream. We found that larger individuals of creek chub and green sunfish were more likely to move, which may be explained by their greater physical ability to balance the energetic cost of moving in tandem with greater competitive ability during settlement. However, the effect of density on movement was mixed, potentially as a result of predator‐prey interactions either directly or indirectly.

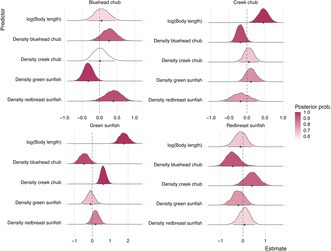

## 1 Introduction

Movement is a ubiquitous characteristic of animals, mediating nutrient exchange, gene flow, and disease spread, among others (Cooke et al. 2022; Hess 1996; Terui et al. 2017; Fausch et al. 2002). If successful, movers may obtain substantial benefits such as release from intense competition and disease risks (Terui et al. 2017; Clobert et al. 2012). However, it comes with energetic and opportunity costs (Bonte et al. 2012). Mobile individuals must make an appropriate decision on when and how they move to improve their fitness, such as growth and survival (Bonte et al. 2012). As a result, movement is influenced by both extrinsic and intrinsic factors, with important consequences for the spatial structure of communities (Leibold et al. 2004; McPeek et al. 2024; Schlägel et al. 2020).

Body size exemplifies intrinsic individual conditions that affect movement patterns (Clobert et al. 2012). For example, larger individuals tend to move long distances due in part to their greater locomotive capabilities, although the nature of correlation varies greatly among species and ecological contexts (Terui et al. 2017; Comte and Olden 2018; Radinger and Wolter 2014; Debeffe et al. 2012; Gilliam and Fraser 2001; de Faustino Queiroz and Terra 2020). Simultaneously, intra‐ and interspecific population densities—key extrinsic factors—influence movement through competitive and mutualistic interactions. For example, competitive interactions may cause individuals to move away from densely populated areas, while mutualistic interactions and higher prey availability may reduce movement rates (Thierry et al. 2024; Rasmussen and Belk 2017; Fronhofer et al. 2018).

Despite this recognition, the interplay between extrinsic and intrinsic factors on movement has rarely been considered (Cooke et al. 2022). Most previous studies have evaluated these factors in isolation due to field constraints and statistical complexity. However, this makes it difficult to understand how observed movement patterns may emerge in nature because these drivers are not mutually exclusive (McMahon and Matter 2006). This unified perspective is particularly important, yet challenging to implement in the field, where multiple species interact and may exhibit species‐specific responses to both intrinsic and extrinsic factors (Terui et al. 2021). Addressing this complexity in movement ecology requires a tractable system, where one can directly observe movement processes while maintaining relevance to natural communities.

Stream fishes serve as an excellent model for evaluating how an intrinsic (body size) and extrinsic driver (population density) shape movement patterns. In a small stream, their movement is restricted to a one‐dimensional system (either up‐ or downstream movement), making the direct observation of movement processes highly tractable in the wild. In addition, as individuals of various sizes move across habitat patches, they engage in inter‐ and intraspecific interactions over space and time (Brown and Lawson 2010; Davidson et al. 2012; Albanese et al. 2004; Nakayama et al. 2018). Thus, fine‐scale movements play a pivotal role in shaping behavioral interactions (Pike and Burman 2023). This suggests that movement, as it persists in nature, is inherently complex; but by evaluating co‐occurring drivers, we can better grasp how such patterns prevail.

Here, we aim to evaluate movement patterns of stream fishes in response to body size and population density. To this end, we conducted a mark‐recapture study in a small stream in North Caroline over a 4‐year period, focusing on four dominant fish species in this system: *Semotilus atromaculatus* creek chub, *Nocomis leptocephalus* bluehead chub, *Lepomis cyanellus* green sunfish, and *L. auritus* redbreast sunfish. These species are largely generalist invertivores, with occasional predation upon other fish species and all begin spawning in the spring. We hypothesize that both intrinsic and extrinsic factors influence the movement of these stream fishes. Specifically, we tested the following predictions: (1) larger individuals move more often than their smaller counterparts; (2) higher interspecific densities will drive more movement due to competition; and (3) higher intraspecific density will drive more movement among conspecifics.

## 2 Method

### 2.1 Study Site and Species

**FIGURE 1:**
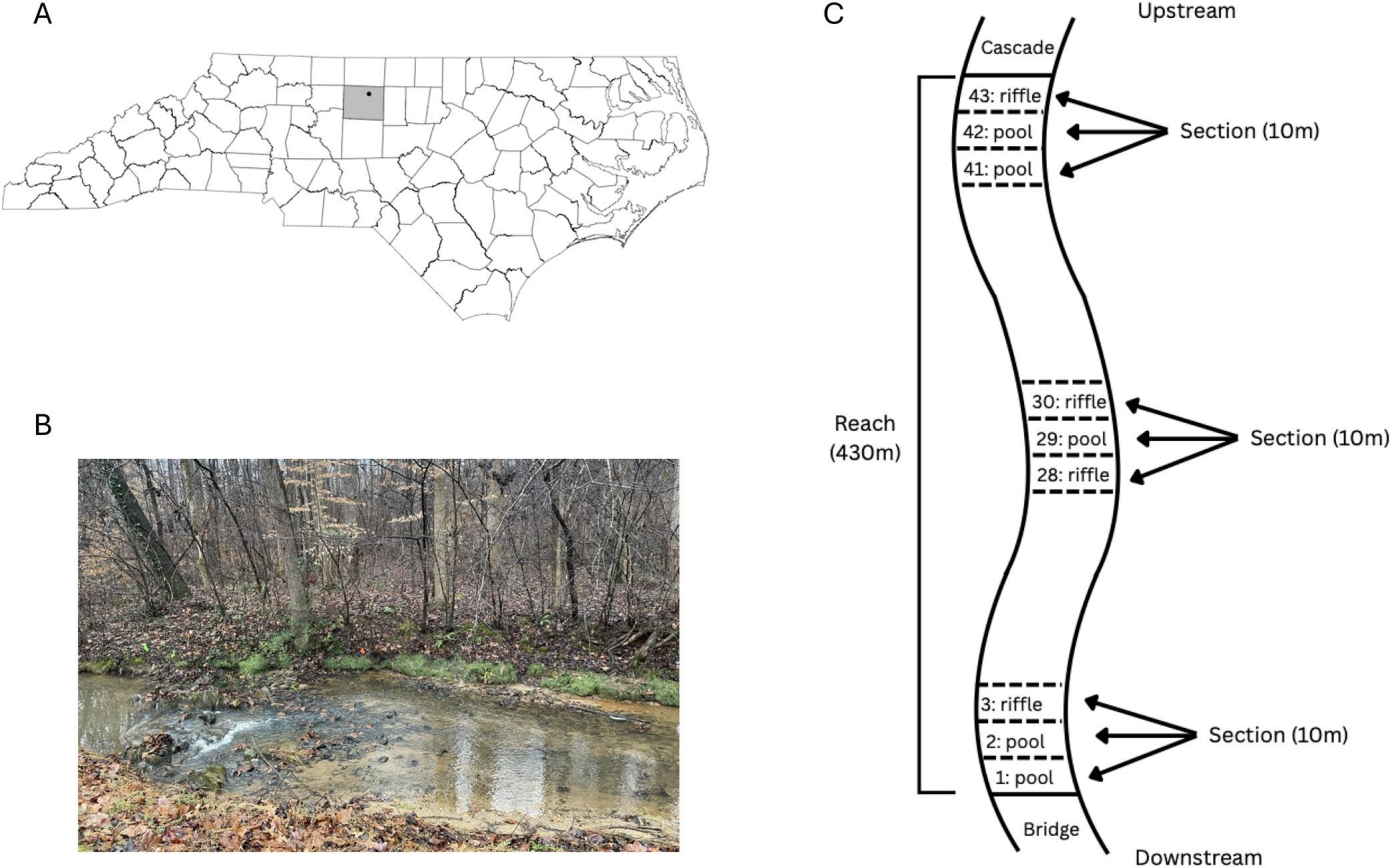
(A) The location of the stream reach within the Piedmont of North Carolina, USA. (B) A picture of the stream for reference. (C) A schematic of the stream reach. Mark‐recapture was conducted in the 430 m reach, broken down by 10 m sections. Sections consisted of pool‐riffle sequences. Each section was sampled using single‐pass backpack electrofishing.

Our study was conducted in the Piedmont region of North Carolina, USA within a small tributary of the Reedy Fork River located at Gateway Research Park (36.169939 N, 79.722088 W). This stream was considered a second‐order perennial from which we selected a 430‐m reach for our mark‐recapture research. This reach consisted of riffle‐pool sequences with its substrate dominated by sand and gravel (Figure 1). The downstream boundary of our study reach ended at a concrete bridge, while the upstream boundary ended at a small cascade.

We selected the four most abundant fish species throughout the reach, all of which generally prefer pool habitats and exhibit resident life‐history strategies (64% in cumulative abundance): two belonging to the Family Leuciscidae ( *Semotilus atromaculatus* creek chub [29% in abundance] and *Nocomis leptocephalus* bluehead chub [14% in abundance]) and two belonging to the Family Centrarchidae ( *Lepomis cyanellus* green sunfish [14% in abundance] and *L. auritus* redbreast sunfish [7% in abundance]).

Creek chub and bluehead chub are morphologically similar but differ slightly in their habitat preferences. Creek chub are most tolerant of environmental degradation and have little preference over habitat (Bramblett et al. 2005; McCormick et al. 2001). Bluehead chub are more selective and prefer gravel and pebble substrate for nest building during the spring spawning season (Evelyn et al. 2024). Despite these subtle differences, habitat requirements of creek and bluehead chub change across body size such that larger individuals are more likely to occupy areas with greater microhabitat availability (Schlosser 1988; Magnan and FitzGerald 1984). Both green and redbreast sunfish are tolerant of a broad range of habitat quality and are nest‐builders during spring spawning (Werner and Hall 1977; Helms et al. 2018; Etnier and Starnes 1993). Largely, these four species are generalist insectivores, but they will occasionally prey upon other fish as adults (Terui et al. 2021; Werner and Hall 1977; Helms et al. 2018; Etnier and Starnes 1993). Proportional abundance for each of these species can be found in Figure S1.

### 2.2 Fish Sampling

Mark‐recapture sampling took place regularly from November 2020 to August 2024 at an interval of roughly 3 months (average 93 days), except for one interval (November 2021 to May 2022; 171 days) in which we were unable to conduct a field survey in February 2022 for logistical reasons. As a result, we collected mark‐recapture data for 15 occasions.

The study reach was divided into 10‐m sections to locate fish at a fine‐spatial scale. In each section, fish were collected via single‐pass electrofishing (Smith‐Root Inc.) and placed in a five‐gallon bucket for handling. All captured individuals were identified to species and measured for total length (mm). Individuals of the study species (> 60 mm in total length) were anesthetized with MS‐222 (Tricaine‐S) and implanted with a 12‐mm passive integrated transponder (PIT) tag to uniquely identify each individual in subsequent recaptures (Oregon RFID). This technique is widely used for fish mark‐recapture in streams, which has been described in more detail by Cary et al. (Cary et al. 2017). After the successful implantation, fish were returned to their holding bucket and monitored for about 15 min to ensure survival before being released back into their section of capture. Across the 15 occasions and four species, we tagged 2430 unique individuals (Table 2).

### 2.3 Environmental Variables

Habitat variables were measured at base‐flow conditions along three evenly spaced transects per section. In each transect, we measured the following physical variables at the center and near both sides of the bank, totaling nine measurements per section: water depth (nearest cm), current velocity (ms^−1^), and dominant substrate type (silt, < 0.1 mm; sand, 0.1–2 mm; gravel, 2–16 mm; pebble, 16–64 mm; cobble, 64–256 mm; boulder, 256–512 mm; bedrock, > 512 mm). In addition, we measured the total aerial coverage of habitat refuge areas (HRA; m^2^) like undercut banks and woody debris per section because these structures may represent important microhabitats for the study species. We calculated the surface area of each section as the mean wet width (measured at each transect) times the section length. Average habitat metrics can be found in Table 1.

**TABLE 1:** Mean and SD across sections and occasions for each habitat variable. Substrate was categorized 1–7 as follows: (1) silt (< 0.01 mm), (2) sand (0.1–2 mm), (3) gravel (2–16 mm), (4) pebble (16–64 mm), (5) cobble (64–256 mm), (6) boulder (256–512 mm) and (7) bedrock (> 512 mm).

Habitat metric	Mean	SD
Mean section area (m^2^)	32.51	9.21
Mean section width (m)	3.26	0.82
Habitat refuge area (m^2^)	0.45	0.84
Mean substrate	4.20	1.42
Mean depth (cm)	18.78	10.31
Mean velocity (m/s)	0.06	0.08

### 2.4 Statistical Analysis

We used the dispersal‐observation model to evaluate the effects of both intrinsic and extrinsic variables on movement behaviors (Terui et al. 2017, 2021; Terui 2020; Rodríguez 2002; Fujiwara et al. 2006). This modeling approach fundamentally differs from ordinary linear models (e.g., generalized linear models) in two key ways. First, it integrates movement and observation processes into a single model. In field studies, observed movement is often confounded by imperfect detection and unmeasured emigration or mortality. The dispersal‐observation model yields less biased estimates of movement parameters by explicitly accounting for these factors (Terui 2020). Second, this framework models the *dispersion* parameter of the movement kernel, i.e., the spread of movement distances, as a function of linear predictors. This contrasts with conventional linear models, which typically relate predictors to the *mean* of a probability distribution. As a result, this approach more appropriately captures variation in movement behavior across ecological contexts (Terui 2020; Rodríguez 2002; Pépino et al. 2012).

#### 2.4.1 Movement Process

We define movement as fine‐scale shifts over habitat patches that occur between consecutive recapture events (i.e., movement between occasion t and t+1). Let $X_{1,i}$ and $X_{0,i}$ denote locations of recapture at occasion t+1 and capture at occasion t for individual replicate i, which were measured as the distance from the midpoint of the section to the downstream end of the study stretch. We assumed $X_{1,i}$ as a random draw from a Student‐t distribution conditional on the capture location $X_{0,i}$ as:

(1) $$X_{1,i}|X_{0,i},\sigma_{i}∼t\left(X_{0,i},\sigma_{i}^{2},\nu \right)$$

where $\sigma_{i}$ is the standard deviation describing the distance moved between consecutive capture and recapture occasions ($X_{1,i}-X_{0,i}$), and ν is the degree of freedom. We chose a Student‐t distribution because movement data are often heavy‐tailed (Clobert et al. 2012). The degrees of freedom parameter, ν, controls the extent of heavy‐tailedness, with lower values indicating fatter tails. As ν approaches infinity, the Student‐t distribution converges to a normal distribution. Here, we selected a moderate value of ν=5 to ameliorate the effect of outliers, such that the coefficient estimates of linear predictors remain relatively insensitive to them (Lunn et al. 2012).

We linked the standard deviation of movement distance to predictors using a log‐link function:

(2) $$\ln\sigma_{i}=\beta_{0}+\sum_{k}\beta_{k}x_{k,i}+\ln\eta_{i}$$

where $\beta_{0}$ is the intercept and $\beta_{k}$ is the regression coefficient for the *k*th predictor $x_{k,i}$. The log‐transformed time interval between capture and recapture $\ln\eta_{i}$ [ln day] was included as an offset term to standardize the movement duration between observations.

Our model included the following predictors at capture ($x_{k}$ in Equation 2): body size, weighted densities of dominant fish species (bluehead chub, creek chub, green sunfish, and redbreast sunfish), Julian day, HRA, and current velocity. We included body size at capture as a proxy variable for the individual's locomotive capacity (internal factor), whereas the weighted fish densities were included to evaluate intra‐ and interspecific density‐dependence in movement (external factors). Fish densities at the capture occasion were weighted by distance to the capture location given that fish individuals in close proximity may have a greater influence on movement. We performed this distance weighting because fish individuals might sense fish densities within a certain distance range, perhaps beyond the capture section. Specifically, the distance‐weighted fish density at section s, denoted $D_{s}^{\left(w\right)}$, was calculated as $D_{s}^{\left(w\right)}=\sum_{s^{\prime }}D_{s^{\prime }}\exp\left(-\lambda d_{ss^{\prime }}\right)$, where $D_{s^{\prime }}$ is the fish density corrected for season‐specific imperfect detection (see Supporting Information: Detection Model and Table S1), $d_{ss^{\prime }}$ is the separation distance between the midpoints of sections s and $s^{\prime }$, and λ is a rate parameter that defines how quickly the density weight decays with increasing separation distance. We set λ=0.1, indicating that density influences are strongest within approximately 10 m, while still allowing for non‐negligible contributions from greater distances. We chose this value because previous studies have consistently shown that stream fishes typically remain within 10–20 m over extended periods, although some individuals do disperse over longer distances (Radinger and Wolter 2014; Terui et al. 2021; Rodríguez 2002; Pépino et al. 2012; Skalski and Gilliam 2000).

Julian day was included to account for potential seasonality in movement patterns. For example, it may capture, albeit implicitly, increased movement due to seasonal spawning behaviors. HRA and current velocity were incorporated to control for possible effects of microhabitat structure variation between sections. HRA may characterize microhabitats that harbor a considerable number of individuals. Current velocity distinguishes pools, runs, and riffles. The inclusion of these predictors allows the model to evaluate the effects of body size and fish densities after accounting for potential influences of confounding factors. We normalized HRA and current velocity by dividing them by their cross‐sectional means calculated for each occasion. This normalization helps properly account for spatial habitat effects on movement processes by removing seasonal differences in their means.

All predictors except for current velocity and HRA were standardized to a mean 0 and a standard deviation 1 prior to the statistical analysis.

#### 2.4.2 Observation Process

Our capture‐recapture data is imperfect representation of movement processes because fish are recaptured only when the following conditions are satisfied simultaneously: alive, stay, and detected in the study section. Our observation model accounts for this process by describing recapture state $Y_{i}$ (1 if recaptured 0 otherwise) as random draws from a Bernoulli distribution:

(3) $$Y_{i}∼\text{Bernoulli}\left(\phi z_{i}\right)$$

where ϕ is the product of survival, detection, and tag‐retention probabilities (hereafter, “recapture” probability) and $z_{i}$ is the binary latent variable indicating whether individual i stayed in the study section at the recapture occasion. The latent variable $z_{i}$ was determined by the observed (if recaptured) or predicted location (if not recaptured) of individual replicate i as:

(4) $$z_{i}=\left\{\begin{array}{c} 1\;\text{if}\;\text{0}\;\leq X_{1,i}\leq L\;\left(\text{stay}\right),\\ 0\;\text{otherwise}\;\left(\text{emigrate}\right). \end{array}\right.$$

L is the upstream terminal of the study reach (L=430). When $X_{1,i}$ was unobserved (i.e., not recaptured), a predicted value was drawn from Equation (1) through the Markov Chain Monte Carlo simulations (see below), thus accounting for the observation process when estimating the movement parameter $\sigma_{i}$. This coupling of observation and movement processes accounts for permanent emigration and yields less biased estimates of movement parameters (Terui 2020). In addition, by including unrecaptured individuals as a recapture state ($Y_{i}=0$), the model can still use the partial movement information from those not recaptured.

The model was fitted to the data for each species separately using JAGS version 4.3.1 (Plummer 2003). Weakly informative priors were assigned to parameters: $\text{Normal}\left({0,2.5}^{2}\right)$ for intercept $\beta_{0}$, $\text{Normal}\left(0,1^{2}\right)$ for coefficients $\beta_{k}$, and $\text{Trunc}\left(\text{Normal}\left({0.5,1}^{2}\right),0,1\right)$ for recapture probability ϕ. Markov chain Monte Carlo (MCMC) simulations were run for 40,000 iterations with a 15,000 burn‐in period and we retained 1000 samples per chain by thinning every 30 steps to calculate posterior probabilities. Model convergence was checked by ensuring that the potential scale reduction factor, referred to as R‐hat, was less than 1.1 for all parameters. All statistical analyses were conducted in R version 4.4.0 (R Core Team 2021).

## 3 Results

Across 15 sampling occasions, we tagged 2430 unique individuals of our target species, 273 of which were recaptured at least once in consecutive surveys (Table 2). Absolute movement distances ranged from 0 to 390 m across species and seasons (Figure S2). For tagged individuals, redbreast sunfish exhibited the largest mean body size (96.2±24.4 mm), followed by creek chub (92.4±21.1 mm), bluehead chub (91.4±21.0 mm), and green sunfish (84.6±18.4 mm) (body size of recaptured compared to unrecaptured can be found in Figure S3). Densities were highest among the creek chub (0.62±0.56 individuals/m^2^), followed by green sunfish (0.51±0.50), bluehead chub (0.31±0.34), and redbreast sunfish (0.29±0.49).

**TABLE 2:** The number of unique individuals tagged, unique recaptures, captured replicates (including multiple captures of the same individual), and recaptured replicates (including multiple recaptures of the same individual) listed from left to right for each target species.

Species	Unique	Unique recaptures	Replicate captures	Replicate recaptures
Bluehead chub	479	73	615	82
Creek chub	1191	111	1391	145
Green sunfish	503	56	612	60
Redbreast sunfish	257	33	317	37

**FIGURE 2:**
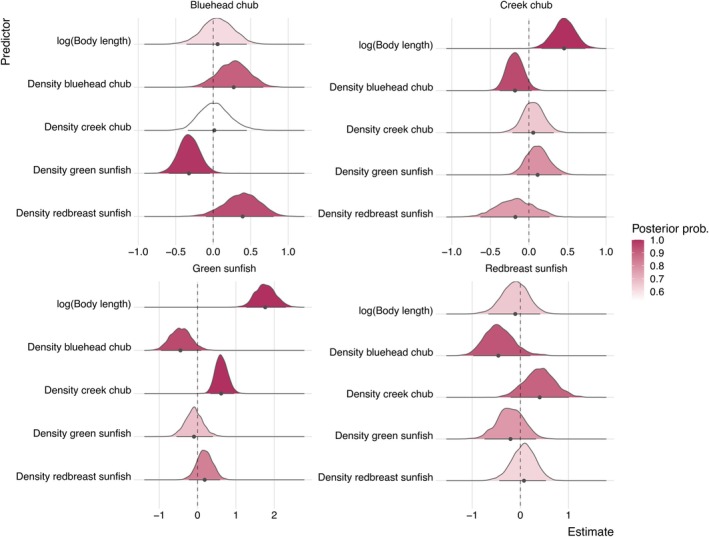
Posterior distributions for parameters in the dispersal‐observation model. Median estimates are denoted by points with error bars showing the traditional 95% credible interval. Distributions are colored in proportion to the posterior probability of each parameter (approximated as the proportion of MCMC samples above or below zeros). Higher values of the posterior probability indicate statistically robust effects. Note that our model also included Julian day, habitat refuge area, and current velocity to minimize potential bias in estimating the effects of body size and fish densities. Full parameter estimates are provided in Table S2.

**FIGURE 3:**
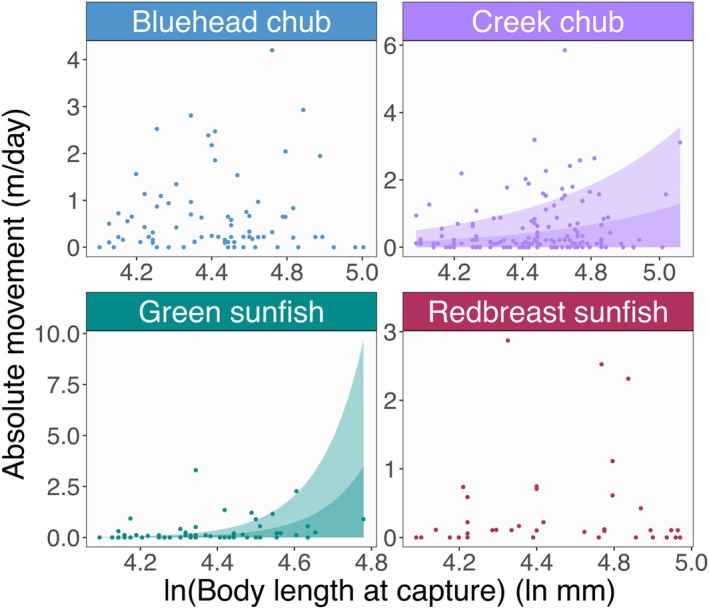
Influence of body size (total body length at capture) on movement distance. Panels and colors distinguish species. Points represent observed values for each recaptured individual. Dark and light shades indicate movement ranges that are predicted to encompass 50% and 90% of individuals by the observation‐dispersal model. Shades are not shown when the posterior probability of the body size effect was less than 0.95. Full parameter estimates are provided in Table S2.

Our model analysis revealed that movement responses to body size and density were highly species‐specific (Figure 2). Creek chub and green sunfish increased movement distance with their body size, and their posterior probabilities were greater than 0.95 (Figures 2 and 3; see also Table S2). However, the movement patterns of bluehead chub and redbreast sunfish showed vague relationships with body size, with the posterior distributions centered around zero (Figure 2).

**FIGURE 4:**
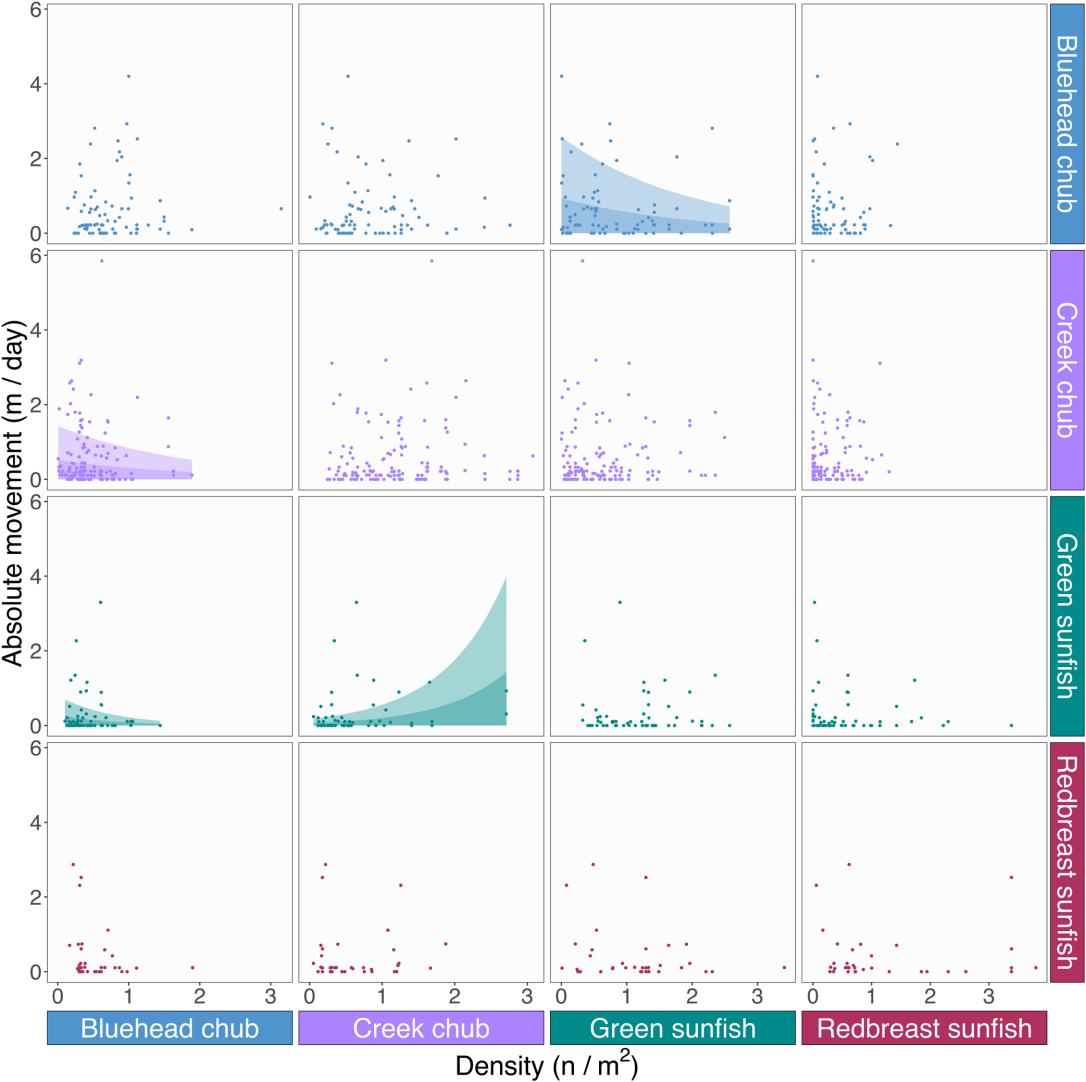
Influence of density on movement distance. Rows represent species responding to intra‐ and interspecific densities, while columns indicate the species whose population densities influence movement. Each panel describes the absolute movement of a species in response to the density of a given species. Colors correspond to species. Diagonal panels show the response to intraspecific density, while the off‐diagonal panels indicate the response to interspecific densities. Points represent observed values for each recaptured individual. Dark and light shades indicate movement ranges that are predicted to encompass 50% and 90% of individuals by the observation‐dispersal model. Shades are not shown when the posterior probability of the density effect was less than 0.95. Full statistics are provided in Table S2.

Interspecific density‐dependence in movement was highly species‐specific (Figures 2 and 4). Green sunfish responded to both creek and bluehead chub, but in contrasting ways. Posterior distributions indicated that green sunfish responded positively to the density of creek chub, suggesting that these species moved away from areas with high population density of creek chub. In contrast, green sunfish responded negatively to bluehead chub such that movement declined in areas with high population density of bluehead chub. Similarly, the posterior distributions indicated that bluehead chub responded negatively to green sunfish density, causing movement to decline when green sunfish density was high. Lastly, creek chub movement was reduced when bluehead chub density was high. Interestingly, no species showed strong evidence of intraspecific density dependence in movement (Figures 2 and 4).

The effect of Julian day on movement was only present in green sunfish, suggesting movement declines as the year progresses into fall and winter. The effects of environmental variables (HRA and velocity) were significant only in redbreast sunfish, with their movement declining as HRA and current velocity increased. Creek chub also tended to move less when current velocity was high, but this effect was marginal. These results were summarized in Table S2.

## 4 Discussion

Movement is shaped by species responses to extrinsic and intrinsic drivers (Clobert et al. 2012). Yet, these factors are often studied in isolation, causing a lack of quantitative analysis comparing their relative influences. Our 4‐year data set of mark‐recapture research unveiled that both intrinsic (body size) and extrinsic (population densities) factors influence stream fish movements, but their influences varied among species. While this makes our findings difficult to generalize, species‐level movement responses can provide greater insight into how species interactions are distributed through non‐random movement.

Our first prediction of size‐dependent movement was supported by creek chub and green sunfish. Movement requires great energetic expenditure that needs to be balanced among individual's needs (Cooke et al. 2022; Boisclair and Leggett 1989; Jobling 1993). Body size is one such limiting factor in determining metabolism (Beamish 1978; Rubio‐Gracia et al. 2020). Because larger individuals have lower relative maintenance costs than smaller individuals due to greater lipid reserves (Brown and Braithwaite 2004; Krause et al. 1998; Kanno et al. 2023), they may be better able to balance the cost of moving (Schlägel et al. 2020), such as traversing across stream sections with swift currents. Not only are larger individuals at an energetic advantage, but they may serve as a stronger competitor in the settlement phase (Rasmussen and Belk 2017). However, size‐dependent movement patterns can be highly flexible and system‐specific. For example, in South Carolina streams during high‐flow disturbance, creek chub did not exhibit size‐dependent movement, whereas bluehead chub—despite showing no such pattern in the present study—demonstrated increased movement distance with body size (Terui et al. 2021). Therefore, species that showed weak or no relationship between movement and body size in this study (bluehead chub and redbreast sunfish) might still exhibit size‐dependent movement in other stream systems. Comparative studies across multiple stream systems may offer deeper insights into the conditions under which size‐dependence emerges.

Our second prediction—higher interspecific density drives more movement—was supported only by green sunfish, which moved longer distances as the density of creek chub increased. The increased movement of green sunfish by creek chub may be indicative of competition between species. In small streams, like the one utilized here, the diets of our target species are known to overlap significantly (Collar et al. 2009; Lemly 1985; Karr 1981; Leonard and Orth 1986), potentially explaining the mechanism behind our observation. As a consequence of this movement process, green sunfish may move to potentially suboptimal habitats in order to avoid competitive interactions (Thierry et al. 2024; Jacob et al. 2018). Green sunfish in particular may be successful in this approach as they are a hardy species that balances the cost and benefit of movement well (Lemly 1985; Moyle et al. 2002). However, creek chub did not move away from areas with higher density of green sunfish, suggesting that this interspecific interaction through movement is asymmetric. This result implies, albeit preliminarily, that creek chub are competitively superior to green sunfish in our study stream.

Contrary to our expectations, however, multiple species tended to stay in areas when interspecific density was high. This pattern was evident for green sunfish and creek chub when the density of bluehead chub was elevated, as well as for bluehead chub when green sunfish density was elevated. The exact mechanism is unclear, but predator–prey interactions might underlie this interspecific density‐dependence (Jacob et al. 2015). Green sunfish have been documented to prey upon various chub species, including bluehead chub (Lemly 1985; Borrelli et al. 2023). In this case, small individuals of bluehead chub may be susceptible to predation by green sunfish. Additionally, bluehead chub build large nests during spawning which create community “hotspots” drawing a diverse array of organisms nearby (Tumolo et al. 2023; Bolton et al. 2015). These bluehead chub nests are built of gravel and pebbles which can provide a microhabitat suitable not only for fish species but for aquatic macroinvertebrates as well (Swartwout et al. 2016). Thus, higher density of bluehead chub may indicate greater food availability, potentially discouraging movement of green sunfish as well as creek chub. Yet, experimental approaches are warranted to elucidate biological causality.

The decreased movement of bluehead chub in response to green sunfish is puzzling, and there are multiple possible explanations for this pattern. First, smaller green sunfish could serve as prey for larger bluehead chub. Yet, despite the omnivorous nature of bluehead chub (Tracy 2009), there is little evidence describing bluehead chub preying upon other fish, making it difficult to envision them preferentially targeting sunfish, which possess spiny fins, over other potential prey items like algae and invertebrates. Second, both species might respond to shared environmental cues, such as increased microhabitat availability (Carey et al. 2010). For example, creek chub and redbreast sunfish both traveled shorter distances with increasing current velocity and/or HRA, suggesting that microhabitat suitability can influence movement decisions. However, while this possibility should not be disregarded, neither of these environmental variables was significant in bluehead chub and green sunfish. Lastly, green sunfish might have facilitative interactions with bluehead chub. Green sunfish build nests by fanning their fins over substrates (Thorp 1988). This spawning behavior may increase access to nest‐building materials for bluhead chub. At present, our data is not sufficient to identify likely mechanisms, and controlled experiments should help elucidate this enigmatic relationship.

Interestingly, no species responded significantly to intraspecific densities, countering our third prediction. This result is somewhat surprising given the wealth of studies showing intraspecific competition exceeding interspecific competition in fishes (Webster and Hixon 2000; Ward et al. 2006) and other taxa (Adler et al. 2018; Barabás et al. 2016; Thompson et al. 2020; Chesson and Huntly 1997; Tilman 1982; McPeek 2012). While high conspecific density could impose intensive intraspecific competition, it also increases the likelihood of finding potential mates, dilution effects, and group feeding (e.g., Allee effects) (Courchamp et al. 2008; Gascoigne and Lipcius 2004; Terui et al. 2015). In our system, this may overshadow the relative competition for other resources like habitat and food, thus producing stronger interspecific interactions. Although further studies are needed to illuminate underlying mechanisms, these opposing influences of conspecific density could potentially obscure the influence on movement patterns.

Although our study illuminated interesting patterns, the results must be viewed with caution. First, while we selected extrinsic and intrinsic variables representative of individual movement ability and species interactions, other potential variables (e.g., sex, disturbance) may be playing a role in driving movement. Evaluating movement as it occurs in situ is an ambitious task; as a consequence, not all possible drivers of movement could be evaluated, like any other field study. This limitation could explain why one of the study species, bluhead chub, did not show interpretable movement patterns in response to selected intrinsic and extrinsic variables. Combining field research with controlled experiments may help address this limitation (Fronhofer et al. 2018; Nathan et al. 2008). Second, we experienced relatively low consecutive recapture rates, likely because of the small spatial extent of our study area. Our dispersal‐observation model is best suited for this type of data though, as it accounts for emigration from the 430‐m study reach, imperfect detection, and survival processes to minimize statistical biases arising from this limitation (Terui 2020). Our simulation study found that this modeling approach provides unbiased estimates of movement parameters, even if the proportion of recaptures is low, by explicitly modeling imperfect recapture. Yet, low recapture rates increase the uncertainty of parameter estimates (Terui 2020). Although our results should be qualitatively robust, their effect size must be interpreted carefully. Lastly, we observed “outlier” movers in the data (Figures 3 and 4). However, since our model employed a Student‐*t* distribution (see Equation 1), our statistical inference is insensitive to these outlier values (i.e., conservative) (Lunn et al. 2012). Nevertheless, it is important to recognize that long‐distance movers can play critical roles in ecological processes (Clobert et al. 2012). Future studies addressing this limitation would be particularly valuable in advancing movement research.

Movement mediates how species interact, shaping the dynamics of spatially‐structured communities (Schlägel et al. 2020). Our study demonstrates how intrinsic (e.g., body size) and extrinsic (e.g., population density) drivers can help pinpoint potential movement patterns in freshwater fishes, with variation among species elucidating a potential avenue to explore community dynamics in the future. Extrapolating how non‐random movements, such as those identified in this study, affect species coexistence and the spatial patterns of community organization will be essential in connecting behavioral ecology to spatial theory.

## Author Contributions

**Ashley LaRoque:** conceptualization (equal), data curation (lead), formal analysis (equal), funding acquisition (supporting), investigation (lead), methodology (equal), visualization (lead), writing – original draft (lead), writing – review and editing (equal). **Seoghyun Kim:** investigation (equal), methodology (equal), writing – review and editing (equal). **Akira Terui:** conceptualization (equal), formal analysis (equal), funding acquisition (equal), methodology (equal), project administration (equal), software (equal), supervision (equal), validation (equal), writing – review and editing (equal).

## Funding

This work was based on materials supported by the University of North Carolina at Greensboro, including Dorothy Levis Munroe Research Award, Faculty Internal Award, John O'Brien Ecological Field Award, Start Up Fund.

## Conflicts of Interest

The authors declare no conflicts of interest.

## Supporting information


**Appendix S1:** ece372996‐sup‐0001‐AppendixS1.pdf.

## Data Availability

All data and R code can be found on the Zenodo repository under the following link: https://doi.org/10.5281/zenodo.17870952.
