# Supplementary material for: Body Size and Local Density Explain Movement Patterns in Stream Fishes
Source: Ecol Evol. 2026 Feb 11;16(2):e72996. doi: 10.1002/ece3.72996 (PMC12895090; doi:10.1002/ece3.72996)
Supplement: Supplementary file 1 — Appendix S1: ece372996‐sup‐0001‐AppendixS1.pdf. [file ECE3-16-e72996-s001.pdf]

Appendix for:

Body size and local density explain movement patterns in stream fishes

Ashley LaRoque[1], Seoghyun Kim[1, 2], and Akira Terui[1]

[1]Depatment of Biology, University of North Carolina at Greensboro

[2]Department of Biological Sciences, Kangwon National University

Detection Model

Our capture-recapture data was collected seasonally and fish may be subject to different detection probabilities by season. As such, accounting for detection probabilities is essential to obtain reliable estimates of fish density. We utilized a spatial Cormack-Jolly-Seber (CJS) model [1] to account for seasonal detection probabilities. This model allowed us to estimate seasonal detection probabilities while accounting for permanent emigration and survival.

Observation process – We assumed that the recapture state $Y_{j,t}$ for unique individual j at occasion t ($Y_{i,t} = 1$ if recaptured, 0 otherwise) is a random draw from a Bernoulli distribution:

$$Y_{j,t} \sim \text{Bernoulli}(p_{i,t} r_{j,t} z_{j,t}), \tag{1}$$

where $p_{j,t}$ is the detection probability, $r_{j,t}$ is the binary latent variable indicating the survival state of individual j, and $z_{j,t}$ is the binary latent variable indicating whether individual j remained in the study section.

Our primary interest was to estimate seasonal detection probabilities. We allowed $p_{j,t}$ to vary by season in

a logit scale:

$$\text{logit}\, p_{j,t} = \mu_p + \alpha_p Q_t, \tag{2}$$

where Q_t is a dummy variable discerning winter (coded as 0: November – February) and summer (coded as 1: May – August). This formulation translates into winter detection as $p_{\text{win}} = \text{inv.logit}(\mu_p)$ and summer detection as $p_{\text{sum}} = \text{inv.logit}(\mu_p + \alpha_p)$

State process – Our model accounted for survival and movement processes to obtain less biased estimates of detection probabilities. The survival state in the (spatial) CJS model conditions on the first capture of individual j. Let f_j denote the first capture occasion for individual j. Then,

$$\begin{aligned} r_{j,f_j} &= 1, \\ r_{j,t+1} | r_{j,t} &\sim \text{Bernoulli}(r_{j,t} s_{j,t}), \end{aligned} \tag{3}$$

where $s_{j,t}$ is the survival probability between t and $t+1$. Since our interest was to estimate detection probabilities, we made a simplifying assumption on the survival probability as $s_{j,t} = \psi^{\eta_{j,t}}$, where ψ is the daily survival probability and $\eta_{j,t}$ is the interval (unit: day) between occasion t and $t+1$.

We also accounted for emigration. Let $X_{j,t+1}$ and $X_{j,t}$ denote locations of recapture at occasion $t+1$ and capture at occasion t for unique individual j, which were measured as the distance from the midpoint of the section to the downstream end of the study stretch. As in the main text, we assumed $X_{j,t+1}$ as a random draw from a normal distribution conditional on the capture location $X_{j,t}$ as:

$$\begin{aligned} X_{j,t+1} | X_{j,t}, \sigma_{j,t} &\sim \text{Normal}(X_{j,t}, \sigma_{j,t}^2) \\ \ln \sigma_{j,t} &= \ln \sigma_0 + \ln \eta_{j,t} \end{aligned} \tag{4}$$

where σ_0 is the standard deviation describing the daily distance moved between occasion t and $t+1$ and $\eta_{j,t}$ is the interval (unit: day) between the occasions for individual j. Unlike the main movement model, we

were unable to include predictors for $\sigma_{j,t}$ since predictor values were unavailable when individual j was not recaptured.

The latent variable $z_{j,t}$ was determined by the observed (if recaptured) or predicted location (if not recaptured) of unique individual j as:

$$z_{j,t} = \begin{cases} 1 \text{ if } 0 \leq X_{j,t} \leq L \text{ (stay)}, \\ 0 \text{ otherwise (emigrate)}. \end{cases} \tag{5}$$

L is the upstream terminal of the study reach ($L = 430$). When $X_{j,t+1}$ was unobserved (i.e., not recaptured), a predicted value was drawn from Equation 4 through the Markov Chain Monte Carlo simulations.

The model was fitted to the data for each species separately using JAGS [2]. Vague or weakly informative priors were used for parameters: Unif(0, 1) for survival probability $s_{j,t}$, and Normal$(0, 10^2)$ for the seasonal effect α_p and the daily movement parameter $\ln \sigma_0$. Markov chain Monte Carlo (MCMC) simulations were run for 20,000 iterations with a 1,000 burn-in period and we retained 1,000 samples per chain by thinning every 40 steps to calculate posterior probabilities. Model convergence was checked by ensuring that the potential scale reduction factor, referred to as R-hat, was less than 1.1 for all parameters. All statistical analyses were conducted in R version 4.4.0 [3].

Estimated detection probabilities were summarized in Table S1. We calculated the corrected density N_{cor} as $N_{\text{cor}} = N_{\text{obs}} p_{\text{sum}}^{-1}$ for summer months and $N_{\text{cor}} = N_{\text{obs}} p_{\text{win}}^{-1}$ for winter months, where N_{obs} is the observed fish density calculated by section and species as the number of individuals per unit surface area. N_{cor} was used as predictors in our movement model in the main text.

Figures

Proportional Abundance

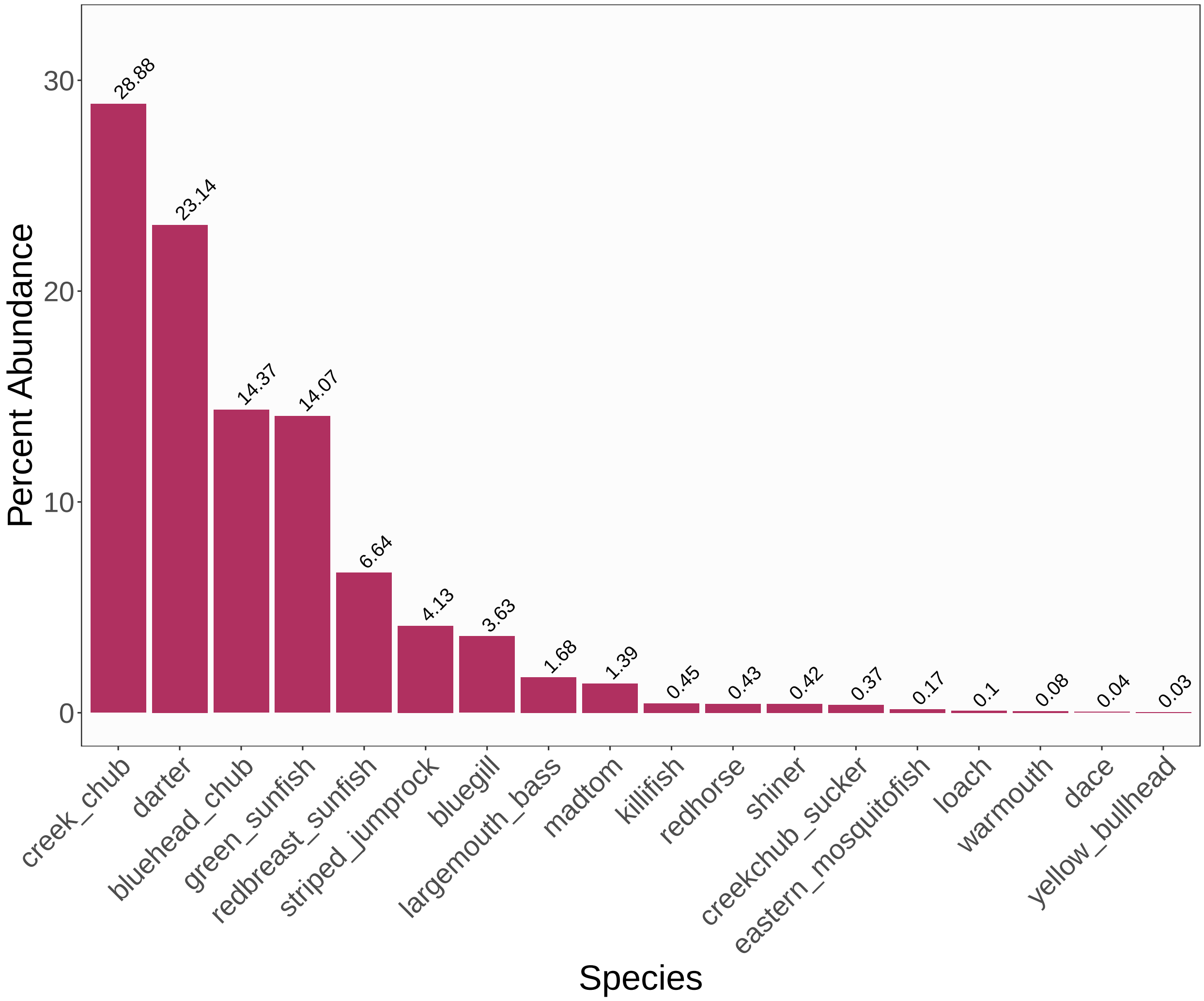

Figure S1: Percent abundance of each species collected during backpack electrofishing ordered from most to least abundant.

Absolute Movement

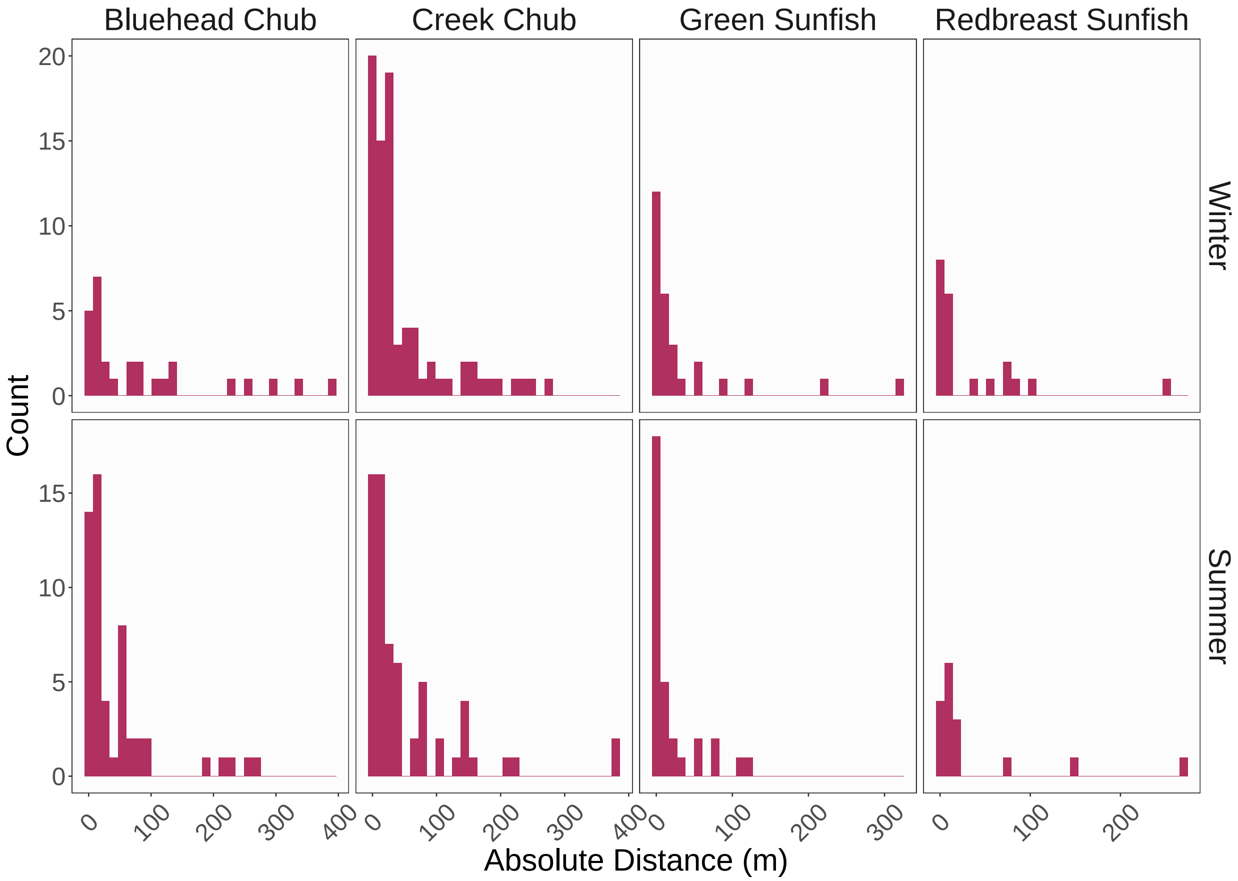

Figure S2: The frequency of absolute movement for each target species shown for both winter and summer seasons. Fine-scale movement is prevalent despite seasonality.

Median Length

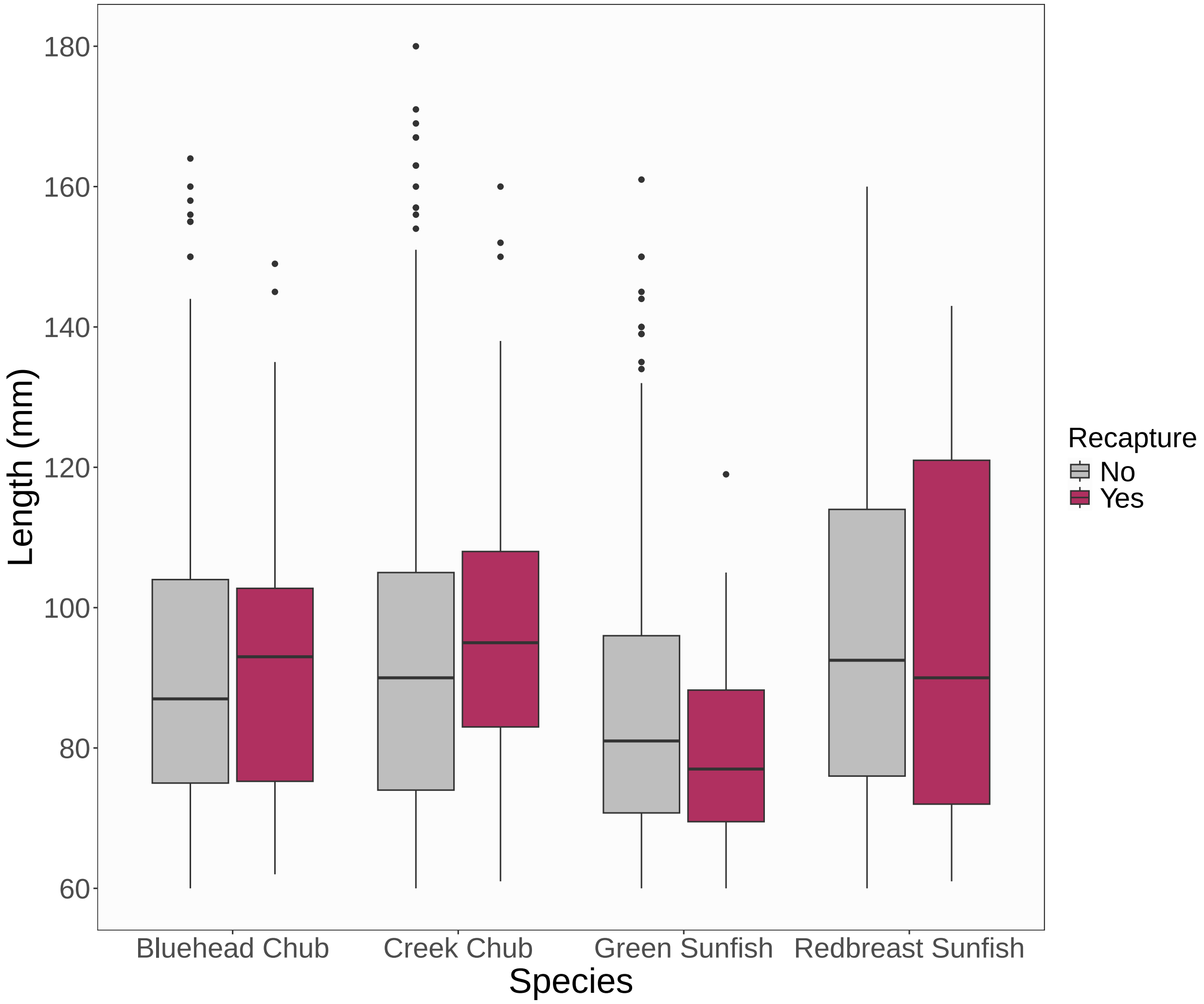

Figure S3: Median length at initial capture for each target species described by the solid black line within the boxplot. Colors indicate whether it is either recaptured (yes) or non-recaptured (no) individuals. The interquartile range is depicted by the size of each box. Outliers are shown as points.

Tables

Seasonal detection probabilities

Table S1: Seasonal detection probabilities estimated using the spatial Cormack-Jolly-Seber (CJS) model, presented as median estimates with corresponding 95% credible intervals in brackets.

Species	Season	Estimate
Bluehead chub	Winter	0.23 [0.18 – 0.30]
	Summer	0.32 [0.25 – 0.41]
Creek chub	Winter	0.25 [0.20 – 0.31]
	Summer	0.35 [0.28 – 0.44]
Green sunfish	Winter	0.13 [0.09 – 0.18]
	Summer	0.21 [0.15 – 0.27]
Redbreast sunfish	Winter	0.12 [0.07 – 0.21]
	Summer	0.29 [0.19 – 0.41]

Movement model coefficients

Table S2: Parameter estimates of the movement model. Median estimates and their associated posterior probabilities are reported.

Species	Effect	Estimate	Pr(< 0)	Pr(> 0)
Bluehead chub	Intercept	0.07	0.41	0.59
	ln(Body length)	0.06	0.39	0.61
	Habitat refuge area	0.03	0.30	0.70
	Current velocity	-0.24	0.80	0.20
	Julian Day	-0.08	0.68	0.32
	Density bluehead chub	0.27	0.10	0.90
	Density creek chub	0.01	0.47	0.53
	Density green sunfish	-0.32	0.99	0.01
	Density redbreast sunfish	0.39	0.06	0.94
	Recapture probability	0.18	0.00	1.00
Creek chub	Intercept	-0.31	0.95	0.05
	ln(Body length)	0.46	0.00	1.00
	Habitat refuge area	-0.02	0.71	0.29
	Current velocity	-0.26	0.96	0.04
	Julian Day	0.13	0.10	0.90
	Density bluehead chub	-0.18	0.95	0.05
	Density creek chub	0.06	0.34	0.66
	Density green sunfish	0.11	0.21	0.79
	Density redbreast sunfish	-0.18	0.76	0.24
	Recapture probability	0.12	0.00	1.00
Green sunfish	Intercept	-1.31	1.00	0.00
	ln(Body length)	1.77	0.00	1.00
	Habitat refuge area	-0.06	0.70	0.30
	Current velocity	-0.20	0.86	0.14
	Julian Day	-0.54	1.00	0.00
	Density bluehead chub	-0.45	0.95	0.05
	Density creek chub	0.62	0.00	1.00
	Density green sunfish	-0.09	0.68	0.32
	Density redbreast sunfish	0.19	0.17	0.83

	Recapture probability	0.12	0.00	1.00
Redbreast sunfish	Intercept	0.77	0.05	0.95
	ln(Body length)	-0.11	0.66	0.34
	Habitat refuge area	-0.59	1.00	0.00
	Current velocity	-0.75	1.00	0.00
	Julian Day	0.23	0.31	0.69
	Density bluehead chub	-0.46	0.92	0.08
	Density creek chub	0.40	0.10	0.90
	Density green sunfish	-0.20	0.77	0.23
	Density redbreast sunfish	0.08	0.38	0.62
	Recapture probability	0.15	0.00	1.00

References

[1] Schaub M, Royle JA. Estimating True Instead of Apparent Survival Using Spatial Cormack–Jolly–Seber Models. Methods in Ecology and Evolution. 2014;5(12):1316-26.

[2] Plummer M. JAGS: A Program for Analysis of Bayesian Graphical Models Using Gibbs Sampling. In Proceedings of the 3rd international workshop on distributed statistical computing. 2003;124(125.10):1-10.

[3] R Core Team. R: A Language and Environment for Statistical Computing. R Foundation for Statistical Computing. 2021;Vienna, Austria.
